# WeatherClean: An Image Restoration Algorithm for UAV-Based Railway Inspection in Adverse Weather

**DOI:** 10.3390/s25154799

**Published:** 2025-08-04

**Authors:** Kewen Wang, Shaobing Yang, Zexuan Zhang, Zhipeng Wang, Limin Jia, Mengwei Li, Shengjia Yu

**Affiliations:** 1 School of Electrical Engineering, Beijing Jiaotong University, Beijing 100044, China; 11350187@chnenergy.com.cn; 2 China Energy Investment Group Co., Ltd. and Xinshuo Railway Co., Ltd. Communications Technology Branch, Ordos 014300, China; 3 School of Traffic and Transportation, Beijing Jiaotong University, Beijing 100044, China; shbyang@bjtu.edu.cn (S.Y.); 24115106@bjtu.edu.cn (Z.Z.); lmjia@bjtu.edu.cn (L.J.); a19912165760@163.com (M.L.); 24120865@bjtu.edu.cn (S.Y.)

**Keywords:** railway safety, UAV inspection, rail perimeter intrusion detection, image de-raining and snow fogging

## Abstract

UAV-based inspections are an effective way to ensure railway safety and have gained significant attention. However, images captured during complex weather conditions, such as rain, snow, or fog, often suffer from severe degradation, affecting image recognition accuracy. Existing algorithms for removing rain, snow, and fog have two main limitations: they do not adaptively learn features under varying weather complexities and struggle with managing complex noise patterns in drone inspections, leading to incomplete noise removal. To address these challenges, this study proposes a novel framework for removing rain, snow, and fog from drone images, called WeatherClean. This framework introduces a Weather Complexity Adjustment Factor (WCAF) in a parameterized adjustable network architecture to process weather degradation of varying degrees adaptively. It also employs a hierarchical multi-scale cropping strategy to enhance the recovery of fine noise and edge structures. Additionally, it incorporates a degradation synthesis method based on atmospheric scattering physical models to generate training samples that align with real-world weather patterns, thereby mitigating data scarcity issues. Experimental results show that WeatherClean outperforms existing methods by effectively removing noise particles while preserving image details. This advancement provides more reliable high-definition visual references for drone-based railway inspections, significantly enhancing inspection capabilities under complex weather conditions and ensuring the safety of railway operations.

## 1. Introduction

In recent years, advancements in drone technology have made railway drone inspections significantly more efficient than traditional methods, particularly in speed and flexibility. However, adverse weather conditions often interfere with drone image acquisition, resulting in blurriness, reduced contrast, and unclear edges. These problems can hinder the drone’s ability to detect foreign objects during inspections. As a result, image enhancement techniques aimed at mitigating weather-related interference—such as rain, snow, and fog—have become a prominent area of research. The primary goal is to restore detailed features within the images, making them more suitable for subsequent analysis and decision-making. Most existing methods focus on eliminating only one type of weather-related noise. The main approaches include vision-based methods [1] and innovative deep learning-based techniques [2,3,4].

UAV-based inspection images pose several challenges, including small inspection targets, varying shooting angles, and complex environments. There is no dedicated algorithm for removing rain, snow, and fog from these images. Furthermore, existing algorithms do not effectively differentiate between various levels of adverse weather, employing a single network for both moderate and extreme conditions. From a network capability standpoint, challenging samples require more parameters, while simpler samples can achieve optimal restoration with fewer parameters. Additionally, current denoising algorithms often fail to eliminate noise particles, resulting in subpar image quality after denoising.

To address these challenges, this paper introduces a noise-supplemented image restoration model with a Dynamic Noise Adjustment Module (DNAM). This module dynamically adjusts its ability to eliminate complex weather effects during inference by modifying the Weather Complexity Adjustment Factor (WCAF). This approach eliminates the need for multiple independent networks while effectively managing various adverse weather conditions, enhancing computational efficiency. Furthermore, to tackle the insufficient handling of fine and residual noise in certain areas by existing denoising networks, this paper employs multi-scale cropping inputs to create multiple loss functions. This strategy improves the model’s capacity to restore local details and effectively reduce noise in small areas, ultimately enhancing overall image quality. As a result, the model provides more stable and accurate restoration performance in complex weather situations. The main contributions of this paper are as follows:

(1) We propose WeatherClean, the first drone inspection-specific rain, snow, and fog removal framework that successfully processes multi-level weather degradation in a single model through the Dynamic Noise Adjustment Module and Weather Complexity Adjustment Factor.
(2) To address the issues of residual noise and detail loss, we design a hierarchical multi-scale optimization loss function that significantly enhances the restoration ability for fine noise and edge structures.
(3) We develop a physics-driven degradation data generation scheme and construct a rain, snow, and fog degradation dataset that aligns with real-world scenarios.

The rest of this paper is organized as follows. Section 2 describes related work, Section 3 presents the proposed WeatherClean method for rain, snow, and fog removal in images. Section 4 discusses ablation studies, comprehensive experiments of the proposed algorithm and their corresponding results. Finally, Section 5 concludes this paper.

## 2. Related Work

In previous studies, researchers mainly focused on image restoration under single weather conditions such as rain, snow, or fog. For example, Shit et al. [5] proposed the EDD-N network, which combines image defogging and detection for real-time haze image processing; Luo et al. [6] designed the LKD-Net, utilizing dilated large kernel convolutional blocks (DLKCBs) and channel-enhanced feedforward networks (CEFNs) to improve defogging performance. However, these methods lack adaptability to various real-world weather conditions. Current leading weather noise removal techniques are based on end-to-end deep learning frameworks, which directly produce fog-free images through network models without estimating intermediate parameters. These methods mainly fall into two categories: GAN-based and Transformer-based models.

In the GAN-based image restoration field, for snow removal, Jaw et al. [7] proposed DesnowGAN to improve visual quality; Zhang et al. [8] designed an algorithm targeting irregular snowflakes and snow traces, combining a residual generator with a novel Transformer module to restore details. For defogging, Wang et al. [9] improved CycleGAN (DFC-dehaze), using a Dehazeformer-t generator and a local–global discriminator to reduce residual haze; Xu et al. [10] developed an enhanced CycleGAN with an adaptive dark channel prior for unpaired single-image defogging.

Transformer-based methods are becoming more popular because of their strong global modeling abilities. In snow removal tasks, Agbodike et al. [11] introduced the WiT network, which combines Vision Transformer and discrete wavelet transform; Wei et al. [12] used ViT to enhance snow removal accuracy; Lin et al. [13] developed the lightweight LMQFormer, capable of removing coarse snow using a masked query module (MQFormer). In rain removal tasks, Tang et al. [14] utilized the Swin Transformer for rain removal and created a twin network model; Song and Chen [15,16] proposed the Cycle-Derain and Cycle-Attention-Derain methods, respectively, which combine unsupervised attention mechanisms with GANs to manage unpaired data. Regarding general image restoration, the OneRestore algorithm shifts self-attention calculations to the channel dimension through MDTA and GDFN modules to lower the complexity of high-resolution images [17]; Duan et al. [18] improved global and local information capture with bidirectional attention and cross-stream convolution modules; He et al. [1] simplified low-light enhancement to a curve estimation task and integrated a CNN-Transformer architecture for low-complexity denoising.

In recent years, multi-complex weather joint removal networks have emerged. Chen et al. [2] propose a visual method capable of simultaneously removing rain streaks and haze; Siddiqua et al. [3] introduce a multi-domain attention-driven multi-modal conditional generative adversarial network (MACGAN) to enhance visibility. Transformer-based approaches include Valanarasu et al. [4] developing an end-to-end single encoder–decoder model capable of handling various weather degradations; the frequency-guided AIRFormer method proposed in Gao et al. [19]; Sun et al. [20] designing a distributed map attention mechanism that divides degraded features into buckets and uses a self-attention mechanism to capture dynamic range dependencies; Wang et al. [21] proposing a TANet algorithm, whose TAB module with spatial attention blocks can be used to handle various weather degradation patterns; Zhu et al. [22] combining the Transformer architecture, hypernetworks, feature-level linear modulation blocks, and contrastive learning to process various weather degradations using the same parameter set.

However, research on drone detection applications remains relatively limited. Wu et al. [23] proposes a drone-based railway image defogging network that uses structural similarity loss to preserve structural information; Yang et al. [24] suggests a discretization-filtering-reconstruction method for processing railway images. Drone-captured images are typically low in resolution, and existing methods often encounter challenges due to high model complexity and strict hardware requirements, making them difficult to deploy in practice. Additionally, their multi-network integration schemes often overlook the real-time processing needs under actual weather conditions. To address these issues, this paper introduces the Transformer-based WeatherClean method, which can adapt dynamically to real-world weather conditions, achieving real-time rain, snow, and fog removal with low complexity, significantly improving the applicability of railway drone detection scenarios.

## 3. Methods

### 3.1. Overall Framework

Existing multi-weather removal methods typically employ architectures with multiple independent encoders. For instance, the All-in-One [25] method formulates the adverse weather removal problem as:

(1) $$B=D(E_{p}(I_{p}))$$

where B is the restored image, D is the decoder, $E_{p}$ is the encoder specific to weather type p, and $I_{p}$ is the input image with weather type p. This approach requires designing independent encoders for each weather type, leading to high computational complexity and lack of information sharing between weather types.

To address these issues, we propose the WeatherClean, which achieves single-network multi-weather adaptive processing through the introduction of a Weather Complexity Adjustment Factor (WCAF), expressed as:

(2) $$B=W(I_{p},\alpha )$$

where W represents the WeatherClean network, and α is the WCAF parameter used to dynamically adjust the network’s capability to handle different weather complexities. It consists of weather-agnostic encoder and decoder networks, unlike the All-in-One network. The weather type queries are learned along with the parameters of W, thus making the problem setup more generic.

**Figure 1:**
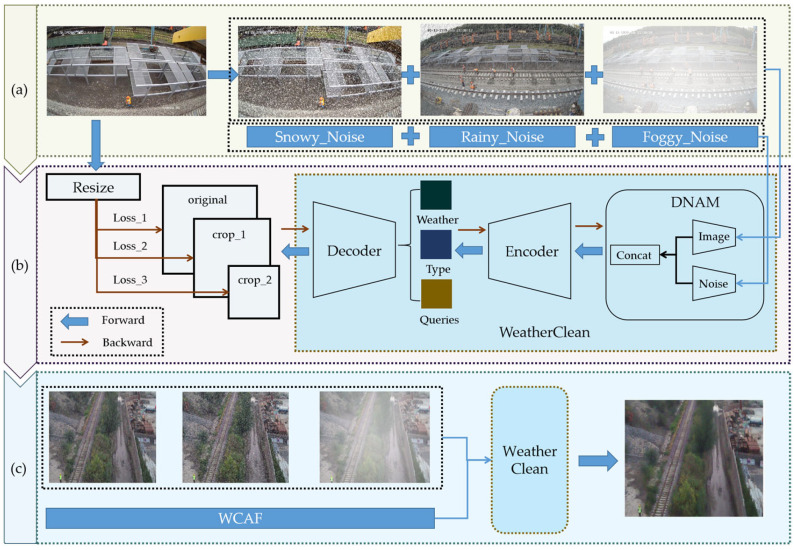
Overall framework of WeatherClean. (**a**) The data processing stage; (**b**) the network training stage; (**c**) the inference stage. The network takes degraded images and the WCAF as inputs and outputs denoised images.

The overall framework of WeatherClean is shown in Figure 1. During data processing, synthetic degraded data are created using physics-based methods, including degraded images and noise as network inputs, with original images used as labels to guide network training. In the training process, multi-scale cropping inputs are used to build multiple loss functions, which improve the model’s ability to restore local details and effectively reduce fine-grained noise, leading to better overall image quality. In the inference stage, the WCAF is employed to enable adaptive processing of multi-level weather degradation.

The remaining sections of this chapter will, respectively, explain how the Dynamic Noise Adjustment Module and the integrated WCAF enhance and guide the network’s adaptive processing of multi-level weather degradation, the WeatherClean network architecture, and the design of the loss function.

### 3.2. The Dynamic Noise Adjustment Module

The denoising process aims to eliminate noise that is not present in the original image. FFDNet [26] assumes additive white Gaussian noise (AWGN) and takes a noise level parameter (σ) along with the noisy image. However, this single-parameter approach offers limited additional information. To improve this, we suggest using σ-generated noise images, allowing the network to learn the specific features of rain, snow, and fog through known noise image characteristics, thereby easing the learning process for the network.

**Figure 2:**
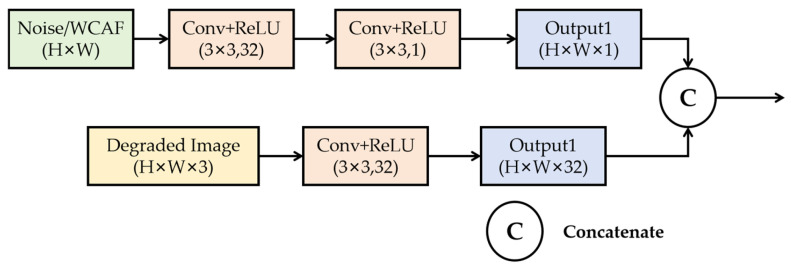
The Dynamic Noise Adjustment Module.

Since the rain, snow, and fog images here are artificially generated by adding noise, if the given prompt information is also Gaussian noise of the same intensity, the network might directly learn the pattern of noise addition from the prompt. To enable the network to learn Gaussian noise of σ intensity, we add two separately generated Gaussian noises of equal intensity, creating a more complex noise distribution in the resulting Gaussian image that conceals its generation method. To incorporate the input noise prompts into the network and better extract information from the noise, we propose the Dynamic Noise Adjustment Module (DNAM), as shown in Figure 2. DNAM employs a 32-channel convolution to project information into a high-dimensional space, while using a 1-channel convolution to compress the information back into a low-dimensional space, since noise information is relatively monotonous compared to image information and requires less data for subsequent processing. The noise information is 1-channel, while the image information is 32-channel, and both are concatenated before passing to the next stage, completing the mixing of image and noise information.

During the inference phase, we introduce the Weather Complexity Adjustment Factor (WCAF) to replace the original noise input, providing more detailed and targeted guidance for the network’s restoration of rain, snow, and fog degradation. DNAM calculates a spatially varying WCAF feature map directly from the degraded image and combines it with high-dimensional image features, allowing subsequent convolutional layers to better perceive and adapt to the severity of degradation at each pixel.

Formally, given a degraded image $I\in {\mathbb{R}}^{H\times W\times 3}$, we first extract three local degradation indicators: contrast C, sharpness L, and noise energy N over sliding windows $\Omega_{ij}$:

(3) $$C(i,j)=\frac{\max_{(u,v)\in \Omega_{ij}}I(u,v)-\min_{(u,v)\in \Omega_{ij}}I(u,v)}{\max_{(u,v)\in \Omega_{ij}}I(u,v)+\min_{(u,v)\in \Omega_{ij}}I(u,v)+\varepsilon }$$

(4) $$L(i,j)=1\;-\;\frac{{\left\|\nabla I\right\|}_{\Omega_{ij}}}{\max{\left\|\nabla I\right\|}_{\Omega_{ij}}+\varepsilon }$$

(5) $$N(i,j)=\frac{\sigma_{\Omega_{ij}}(I)}{\max\sigma_{\Omega_{ij}}(I)+\varepsilon }$$

where ∇I denotes the image gradient, $\sigma_{\Omega_{ij}}(I)$ is the local standard deviation, and ε is a small constant for numerical stability. These three feature maps are concatenated along the channel dimension and passed through a 1 × 1 convolution followed by a sigmoid activation to yield the WCAF map:

(6) $$WCAF=\sigma (W*[C,L,N]+b)\in {\left(0,1\right)}^{H\times W}$$

where * denoting convolution and [C,L,N] are the channel-stacked features.

During the inference stage, the WCAF dynamically adjusts noise intensity to match the degradation complexity in the input image. Specifically, the network creates a complexity factor for each pixel, with values ranging from 0 to 1; higher values near 1 indicate more severe degradation, such as heavy rain or snow, while values near 0 represent mild weather effects like light fog or haze. This dynamic complexity factor allows the network to adaptively modify the denoising strength during noise processing to reflect the actual degradation level. For example, when the WCAF value is high, the noise intensity increases accordingly, helping the model handle severe weather-related image degradation; when the WCAF value is low, the noise intensity decreases, making image restoration easier.

### 3.3. WeatherClean Network Architecture

**Figure 3:**
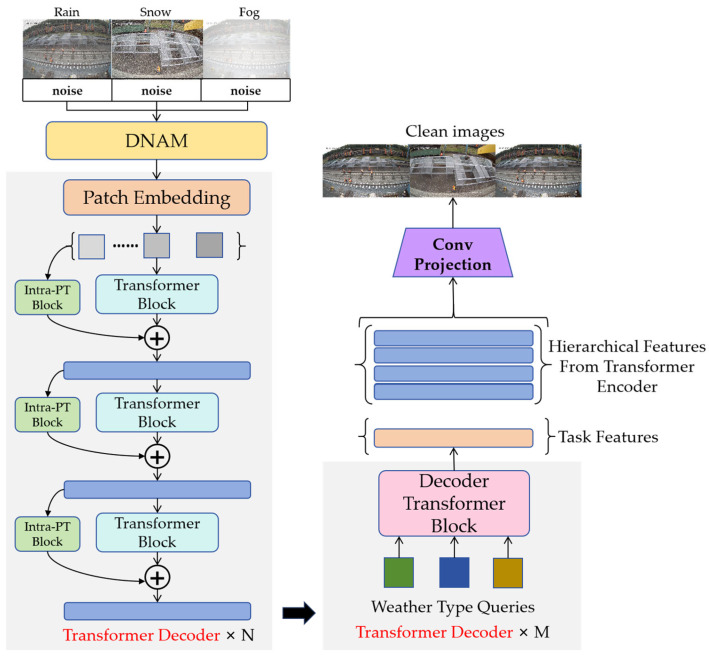
Overview of the proposed WeatherClean network.

Inspired by TransWeather’s single-network approach to multi-weather degradation, we design an innovative model called WeatherClean to simultaneously eliminate image quality degradation caused by three types of weather conditions: rain, snow, and fog. The network architecture of WeatherClean is shown in Figure 3, which mainly consists of a Dynamic Noise Adjustment Module, an encoder, a decoder, and a convolutional projection module. The network first receives input from the DNAM as a processed noisy image of size H × W × 3. Next, the network divides the noisy image into multiple patches and inputs these patches into the encoder of the Transformer module at different processing stages. The resolution of the image is gradually reduced at each stage to ensure that the Transformer can effectively learn both coarse and fine image details. In the decoding stage, the encoded features are used as keys and values within the Transformer decoder block, and the learnable weather type query embedding is used as the query. The convolutional projection block then produces a denoised image with dimensions H × W × 3.

#### 3.3.1. Encoder Design

The encoder is based on the Transformer architecture, which derives hierarchical feature representations of the input image through multi-level processing within the encoder. At various stages, both high-level and low-level image features are extracted. In each stage, an overlapping patch merging strategy is used, where overlapping feature patches are combined into features of the same size as non-overlapping patches before passing to the next stage for further feature extraction.

**Figure 4:**
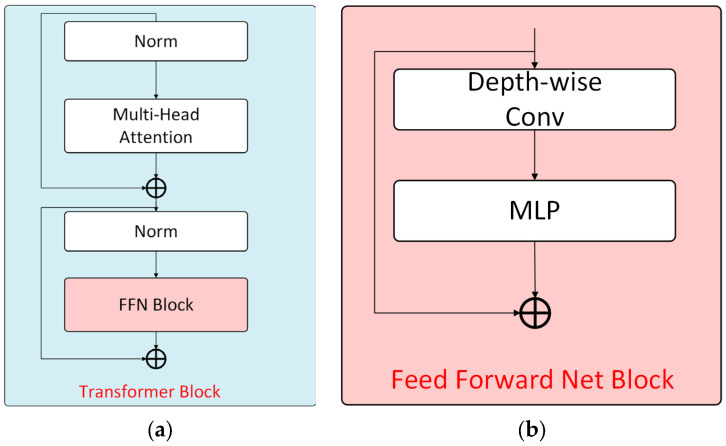
Encoder design. (**a**) Schematic diagram of the encoder module architecture; (**b**) the FFN Module Architecture.

As shown in Figure 4a, each Transformer module includes a multi-head self-attention mechanism and a feed-forward network for computing self-attention features. For each computational stage, the process can be formally expressed as:

(7) $$T\left(I\right)=FFN\left(MSA\left(I\right)+P\right)$$

where T(⋅) denotes the Transformer module, FFN(⋅) represents the feed-forward network, MSA(⋅) indicates the multi-head self-attention network, and P corresponds to the input image.

Similar to the original self-attention mechanism, each head in the multi-head self-attention network maintains identical dimensions for queries (Q), keys (K), and values (V). Assuming d represents the feature dimension, multiple attention heads exist within each encoder module. The number of heads serves as a tunable hyperparameter that is adaptively adjusted across different stages of the Transformer encoder. The attention computation is formulated as:

(8) $$Attention=softmax\left(\frac{QK^{T}}{\sqrt{d}}\right)V$$

To lower computational complexity, a reduction ratio R is introduced, which reduces the original self-attention complexity from $O(N^{2})$ to $O(N^{2}/R)$.

The implementation involves reshaping the key matrix from (N,C) to (N/R,C×R), followed by a linear projection that maps the second dimension from C/R back to C, resulting in adjusted key dimensions of (N/R,C). The processed self-attention features are then propagated to the feed-forward network module. Notably, unlike standard Transformer architectures, the proposed feed-forward network incorporates depthwise convolution to capture local information and enhance positional awareness for the Transformer. The feed-forward network module structure is illustrated in Figure 4b.

Given the self-attention features I as input, where DWC(⋅) denotes depthwise convolution, GELU(⋅) represents the Gaussian Error Linear Unit, and MLP(⋅) denotes the Multilayer Perceptron. The computation in the FFN module is formulated as follows:

(9) $$FFN\left(I\right)=MLP\left(GELU\left(DWC\left(MLP\left(I\right)\right)\right)\right)+I$$

#### 3.3.2. The Intra-Patch Transformer Module

**Figure 5:**
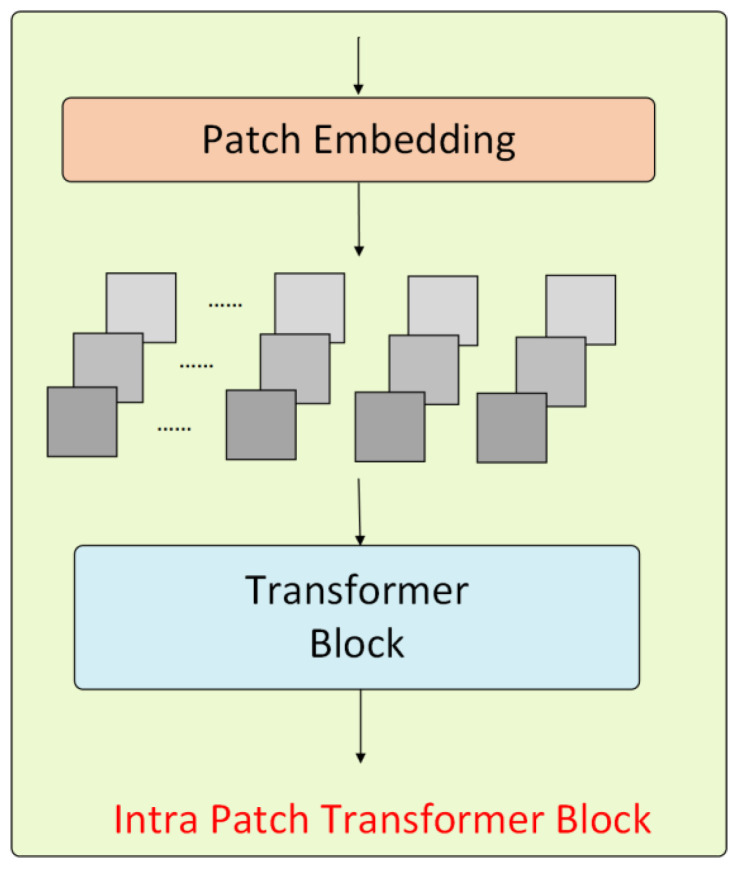
The Intra-PT module structure.

During the forward propagation in the Transformer encoder, the Intra-PT (intra-patch Transformer) module is inserted between each stage to process the sub-patches created from the original patches. The dimensions of these sub-patches are half the width and height of the original patch. The structure of the Intra-PT module is shown in Figure 5.

The Intra-PT module employs a similar Transformer architecture as the main module, but uses a higher reduction ratio R for increased computational efficiency. Because it operates on smaller patches, it can capture finer details, reducing the loss of small-scale information. Except for the first stage, the Intra-PT module generates patches at the feature level, whereas the first stage produces patches at the image level. The output self-attentive features of the Intra-PT module are combined at the same stage as those from the main module’s self-attentive features. Therefore, at each stage of the forward propagation, the Transformer encoder’s process can be summarized as:

(10) $$Y=T_{main}\left(I\right)+T_{IntraPT}\left(SP\left(I\right)\right)$$

where Y is the output of the Transformer at each stage, I is the input of the Transformer at each stage, $T_{main}$ is the main Transformer module, and $T_{IntraPT}$ is the Intra-PT module in the Transformer, which corresponds to the process of creating sub-patches from the input patches.

#### 3.3.3. Decoder Design

Traditional Transformer decoders use autoregression to predict the output sequence one element at a time. Inspired by Detection Transformer (DETR), this method defines weather type queries that are used to decode task feature vectors and reconstruct clear images accordingly. These weather type queries are learnable embeddings, trained alongside other network parameters and associated with the feature output of the Transformer encoder.

**Figure 6:**
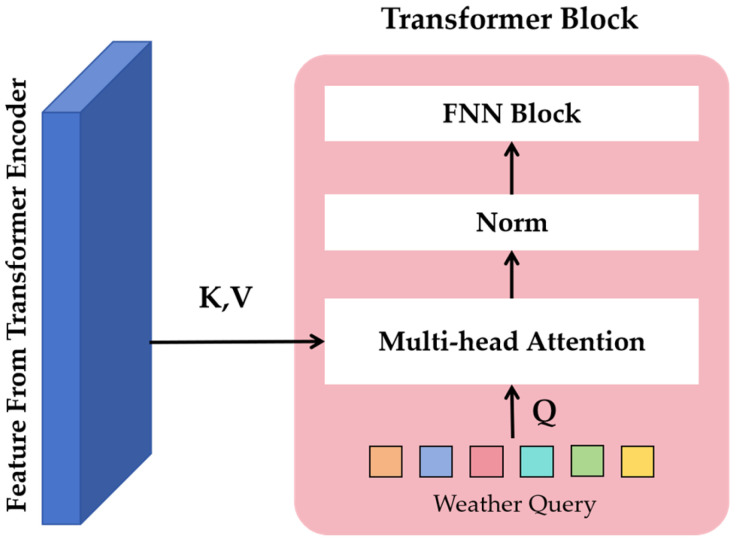
The structure of the Transformer module in the decoder.

The Transformer decoder operates as a single stage but incorporates multiple blocks. Figure 6 shows the structure of the Transformer block in the decoder.

Unlike the Transformer module in the encoder, the query Q in the decoder is a learnable embedding that represents the weather type, while the key K and the value V are features extracted from the final stage of the Transformer encoder. These Transformer modules are similar to those in the encoder–decoder architecture. Unlike the self-attentive Transformer module (where Q, K, and V are all from the same input), Q in the decoder is a weather-type learnable embedding, while K and V are features from the Transformer encoder. The features output from the decoder represents the task feature vector, which is fused with the features extracted by the Transformer encoder at each stage and eventually fed back into the convolutional tail to reconstruct a clean image.

For the convolutional layer projection module design, the layered Transformer encoder feature set and task features from the Transformer decoder are processed through four convolutional layers to produce a clean image. Before each convolutional layer, an upsampling layer is used to restore the original image size. Jump connections are established at each stage of the convolutional tail, starting from the Transformer encoder, and the tanh activation function is applied in the final layer.

### 3.4. Loss Function

**Figure 7:**
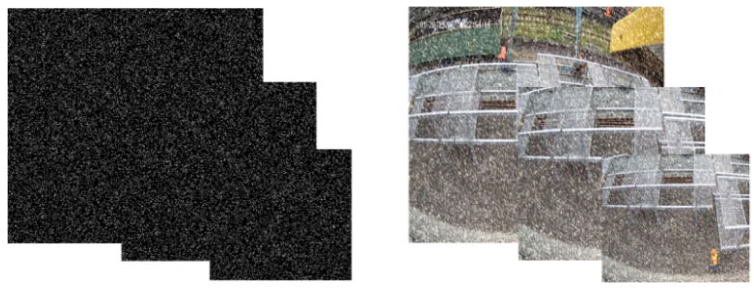
Illustration of multi-scale cropped input patches.

To handle complex weather conditions such as different levels of rain, snow, and fog, the proposed algorithm includes a flexible denoising module capable of managing degradation tasks across various weather intensities. However, this flexibility might cause the network to overfit, which could reduce its performance on single-weather scenarios. To improve the model’s ability to process specific weather conditions, the WeatherClean network uses a multi-scale cropping strategy as an input augmentation method. As shown in Figure 7, this approach takes advantage of the randomness in smaller input patches to enhance the network’s generalization. Additionally, various loss functions are used to regulate the network’s outputs, ensuring strong performance across different weather situations.

The network is trained in an end-to-end manner, with the loss function using a smooth L1 loss function between the predicted and true values. The residual loss between the predicted value and the true value is defined as *f*, and the loss function L1 is defined as follows:

(11) $$Loss_{L1}=\left\{\begin{array}{c} 0.5f^{2},|f|<1\\ |f|-0.5,|f|\geq 1\; \end{array}\right.$$

In order to better enhance the detailed information of the output, the perceptual loss is also used to measure the difference between the predicted features and the real features. These features are extracted using the VGG16 network pre-trained on ImageNet. The features are extracted from layers 3, 8, and 15 of VGG16 to calculate the perceptual loss. The perceptual loss is calculated as follows:

(12) $$Loss_{perceptual}=Loss_{MSE}\left(VGG\left(\hat{Y}\right),VGG\left(Y\right)\right)$$

where $Loss_{MSE}$ is the mean square error loss function, VGC is the feature extraction output from the VGG16 network, Y is the predicted value, and $\hat{Y}$ is the true value.

For the three different sizes of inputs and outputs after dataset cropping, the smoothing L1 loss and perceptual loss are applied to the three predicted and true values, respectively. The different scales used in this algorithm are original size, 128 × 128 and 64 × 64, respectively; the larger size cropped input can capture the overall effect of the whole image and handle the variation in different noise reduction intensities. Meanwhile, the smaller size cropped input with randomization allows the network to focus on details and small noises in the image, significantly improving its noise reduction ability for a single focus area. The total network loss can be expressed as the following equation:

(13) $$Loss_{total}=Loss_{O}+Loss_{c1}+Loss_{c2}$$

where $Loss_{O}$, $Loss_{c1}$ and $Loss_{c2}$ represent the loss functions between the output values and labels of three different sizes after cropping, which can all be expressed by the following formula:

(14) $$Loss=Loss_{L1}+\lambda Loss_{perceptual}$$

λ is a weight controlling the contribution of the smoothed L1 loss and the perceptual loss to the total loss, and is set to 0.04 here.

## 4. Experiments

### 4.1. Experimental Datasets

#### 4.1.1. Datasets

In the study of railway image de-rain, de-snow, and defog, obtaining real-world images with rain, snow, or fog conditions and their corresponding clear images is extremely challenging. Therefore, synthetic datasets have become a primary data source in this research. To address the challenge of removing rain, snow, and fog from railway images and to create a large-scale paired dataset, images from the Kaggle Railroad Worker Detection Dataset [27] are used. Two methods are employed to generate rainy, snowy, and foggy weather conditions.

To simulate the effect of rain and snow on an image, a method based on random sampling and image processing is used. First, random noise regions of the same size as the target image are generated by uniformly sampling pixel values from 0 to 255. The noise distribution is controlled by setting a threshold, and a convolution kernel is applied to initially blur the noise, making it resemble rain and snow in the actual scene more closely. Additionally, a rotation matrix with a specific angle is used to simulate the tilt of the rain and snow, and this matrix is blurred with an equally scaled Gaussian kernel. This not only widens the noise but also makes it more dynamic.

Finally, the blurred rotation matrix is used as a filter to process the initial noisy image. To incorporate rain and snow effects into the original image, we superimpose the noisy image onto the original image proportionally based on pixel values. This results in a synthesized image with the rain and snow effects.

Fog is a dense suspension of tiny water droplets or ice crystals in the atmosphere. Its scattering and absorption characteristics can be described by Mie scattering theory. The presence of fog causes light scattering during transmission, leading to reduced contrast and less saturated object colors. In computer vision, the atmospheric scattering model is commonly used to mathematically represent foggy images, as fog formation is naturally connected to light scattering phenomena in the atmosphere. This scattering process results in the weakening of light by atmospheric particles such as haze and mist. The standard optical model is as follows:

(15) $$I\left(x\right)=J\left(x\right)t\left(x\right)+A\left(1-t\left(x\right)\right)$$

where A is the global atmospheric light component, x is the coordinate value of image pixels, I(x) is the fog image, t(x) is the transmittance, and J(x) is the fog removal image to be restored [27]. According to the formula, the image was processed and the synthetic fog figure was obtained.

The paired dataset was created through various parameter adjustments and split into training and validation sets, with the training set containing 7524 images and the test set including 2142 images. The number of images for rain, snow, and fog conditions is evenly distributed. To further assess the algorithm’s ability to generalize across different scenarios, we built a rain, snow, and fog degradation dataset based on Railway Foreign Object Intrusion images captured by drones. Using the same synthesis method for rain, snow, and fog, we generated a total of 240 synthetic weather images, with 80 images for each weather condition. Additionally, we collected 1372 foggy railway images taken by drones during early morning hours for real-world scenario validation.

#### 4.1.2. Experiment Environment and Parameter Settings

The hardware environment for this experiment consists of an NVIDIA GeForce RTX 4090 Ti GPU, while the software environment includes the Ubuntu 18.04.5 operating system, Python 3.8.5, Pytorch 1.8.0, and relevant deep learning Python libraries. The Adam optimizer is used with a learning rate of 0.0002. A learning rate scheduler is employed to update the learning rate by a factor of 2 after 100 and 150 iterations. The network is trained for 500 epochs, with a batch size of 8 for the training set and a batch size of 1 for the validation set.

#### 4.1.3. Evaluation Metrics

In this experiment, two evaluation metrics, the Peak Signal-to-Noise Ratio (PSNR) and the Structural Similarity Index (SSIM), are used to assess the performance of the proposed algorithm [28]. These two metrics are commonly employed to evaluate the similarity between images, but they focus on different aspects. The PSNR is a widely used metric for assessing image quality. It measures the Peak Signal-to-Noise Ratio between the original image and the image that has undergone compression or other distortion processes. A higher PSNR value indicates better image quality. The calculation formula for the PSNR is as follows:

(16) $$PSNR=10\times \log_{10}(\frac{MAX^{2}}{MSE})$$

where MAX represents the maximum possible pixel value, while MSE is the mean squared error between the original image and the distorted image. The calculation formula for MSE is as follows:

(17) $$MSE=\frac{1}{mn}\sum_{i=0}^{m-1}\sum_{j=0}^{n-1}{\left[I(i,j)-K(i,j)\right]}^{2}$$

where I(i,j) represents the pixel value of the original image, K(i,j) is the pixel value of the distorted image, and m and *n* are the width and height of the image, respectively.

The SSIM is another metric used to evaluate image quality. It considers not only differences in pixel values but also the image’s structure and human visual perception. The calculation formula for the SSIM is as follows:

(18) $$SSIM(x,y)=\frac{\left(2\mu_{x}\mu_{y}+c_{1})\cdot (2\sigma_{xy}+c_{2}\right)}{\left(\mu_{x}^{2}+\mu_{y}^{2}+c_{1})\cdot (\sigma_{x}^{2}+\sigma_{y}^{2}+c_{2}\right)}$$

where $\mu_{x}$ and $\mu_{y}$ represent the mean values of images x and y, respectively, $\sigma_{x}^{2}$ and $\sigma_{y}^{2}$ are their variances, and $\sigma_{xy}$ is their covariance. $c_{1}$ and $c_{2}$ are constants used to stabilize the calculation.

### 4.2. Ablation Study

**Figure 8:**
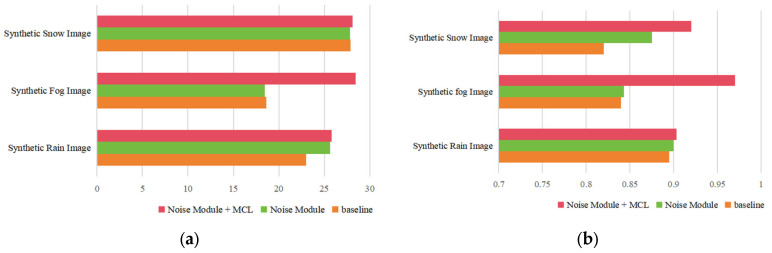
Comparison of the PSNR and the SSIM for different components. (**a**) The PSNR; (**b**) the SSIM.

Through ablation experiments, the two main components of the proposed network in this section, namely the Dynamic Noise Adjustment Module (DNAM) and the Multi-scale Crop Loss (MCL), are validated regarding their impact on the network’s denoising performance using the Railway Public Synthetic Dataset. To quantitatively assess the denoising performance of this method, two metrics are introduced: the PSNR and the SSIM. The results of ablation experiments on synthetic railway public datasets for rain, snow, and fog are shown in Figure 8 and Table 1.

1. Analysis of the Ablation Experiment Results for the DNAM

The Dynamic Noise Adjustment Module aims to effectively integrate input noise information with the network to enhance noise feature extraction. DNAM and degraded images are simultaneously input into the network, allowing the model to learn rain, snow, and fog characteristics from the noise images provided. As shown in the first and second rows of Table 1, after incorporating DNAM, the rain removal performance is the best, with the PSNR increasing from 22.98 to 25.64 and the SSIM from 0.8107 to 0.8947, representing improvements of 11.6% and 10.4%, respectively. Snow removal results are roughly comparable to the baseline, with a slight increase in the SSIM but a minor decrease in the PSNR. The fog removal performance shows a small decline, with the PSNR dropping from 18.63 to 18.46 and the SSIM from 0.8240 to 0.8233. This indicates that DNAM performs exceptionally well in handling high-frequency noise but has limitations in addressing low-frequency degradation, suggesting that combining it with multi-scale cropping loss functions could further improve results.

2. Analysis of the Ablation Experiment Results for the Multi-scale Crop Loss

When the network is trained to remove noise from degraded images, sometimes too many details are lost in the denoising results. To address this issue, this study proposes a Multi-scale Crop Loss (MCL) function to handle variations in denoising strength, which significantly improves the denoising ability for specific concentrations. In this study, cropped images of sizes 128 × 128 and 64 × 64 are added to the input of the WeatherClean network, with the loss function imposing constraints on the effect. As shown in the third row of Table 1, using the MCL function in the network results in a more effective improvement in rain, snow, and fog removal performance compared to using only the DNAM. As shown in the first and third rows of Table 1, after applying both the DNAM and the loss function, the model’s denoising performance significantly improves compared to the original model, especially in the case of synthetic fog, where the increases in the PSNR and the SSIM are substantial.

3. Analysis of the Adaptation Mechanism of the Improved Modules to Different Weather Types

After adding the DNAM, the most notable improvement was seen in rainy images, where the PSNR increased by 2.66 (from 22.98 to 25.64). This is mainly because the Noise Module effectively uses noise priors to guide feature extraction, removing the linear noise caused by rain. However, the improvement in snowy and foggy images was less obvious. Snow noise is more scattered and granular, so the DNAM performs less well with this type of noise. In foggy images, the overall degradation and the inability of the DNAM to restore contrast limit its effectiveness. Therefore, the DNAM alone does not produce significant improvements in snowy and foggy images.

However, after adding the Multi-scale Crop Loss, the results improved significantly. MCL boosts the model’s ability to recover local details and small-scale noise by using a multi-scale mechanism, especially in snowy and foggy images, where both the PSNR and the SSIM saw notable gains. Specifically, in foggy images, the PSNR reached 28.44, and the SSIM increased to 0.9548, showing that MCL effectively restored both image details and overall contrast.

**Figure 9:**
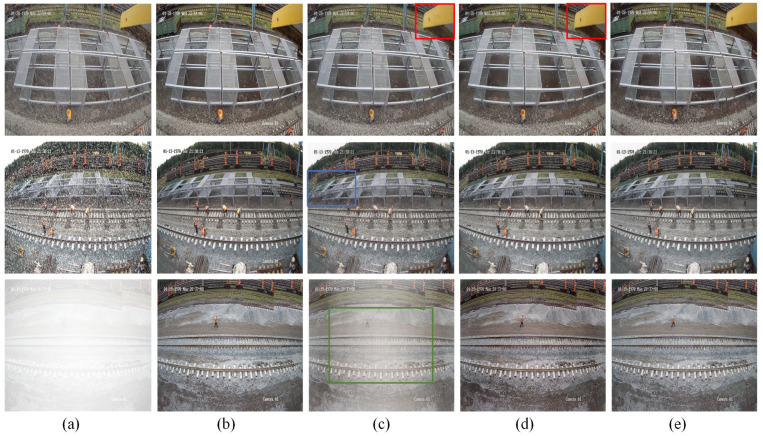
Ablation study visualization results. (**a**) Degraded images; (**b**) real images; (**c**) baseline; (**d**) +DNAM; (**e**) + DNAM+ MCL.

To better demonstrate the results of the model on the Railway Public Dataset, the effects of the DNAM and MCL loss function are visually displayed, as shown in Figure 9. Red boxes indicate rain residue, blue boxes indicate snow residue, and green boxes indicate fog residue. It can be observed that the proposed DNAM and MCL function have a noticeable positive effect on the image quality.

### 4.3. Comprehensive Comparative Experiments

To assess the generalizability of the proposed model, comprehensive comparative experiments were performed on images from the Railway Foreign Object Intrusion Sample Database. The model was evaluated against leading denoising algorithms such as TransWeather [4], MPRNet [29], Restormer [17], Uformer [30], and All-in-One [25]. The results of these comparisons for the rain, snow, and fog synthetic datasets collected by drones are shown in Table 2.

The comprehensive comparative experimental results show that the WeatherClean model performs very well under all weather conditions. Specifically, in rainy conditions, WeatherClean achieves a PSNR of 30.05 and an SSIM of 0.8111, both exceeding all comparison models and showing its excellent ability to handle linear noise. In snowy conditions, WeatherClean has an SSIM of 0.8024, the highest among all models, and a PSNR of 28.49, just below All-in-One’s 28.57, indicating its balanced processing ability. In foggy conditions, WeatherClean stands out most clearly, with a PSNR of 28.50—much higher than the second-best All-in-One at 14.23—and an SSIM of 0.9548, considerably higher than the other models, demonstrating its strong advantage in dealing with low-frequency degradation.

**Figure 10:**
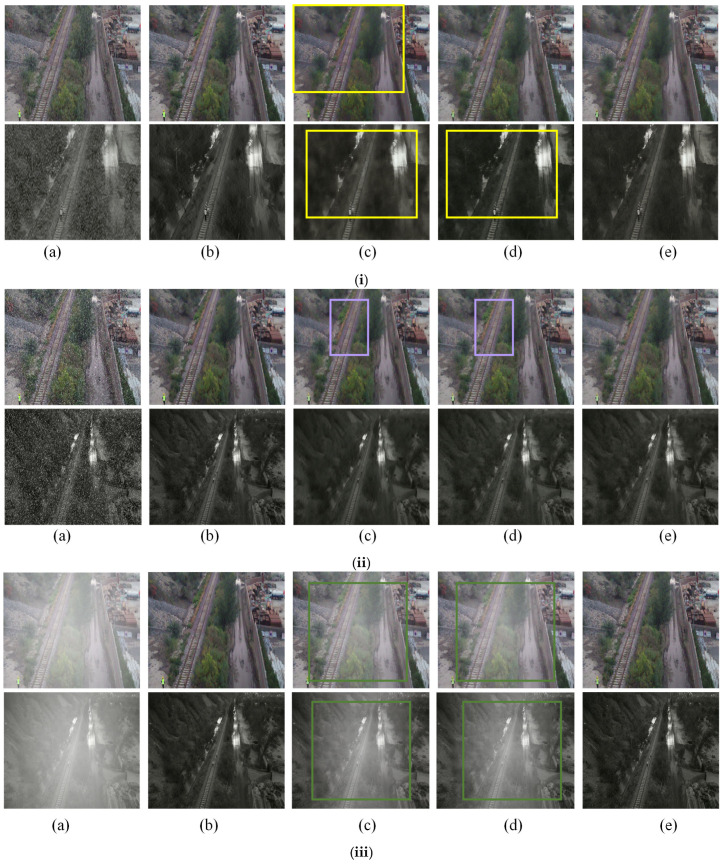
Visualization of image denoising results. (**i**) Rainy condition; (**ii**) snowy condition; (**iii**) foggy condition. (**a**) The degraded image; (**b**) The ground truth (GT); (**c**)The result of MPRNet; (**d**) The result of TransWeather; (**e**) The result of WeatherClean.

The denoising results of the algorithm proposed in this paper are compared with those of current mainstream denoising algorithms, TransWeather and MPRNet. The rain removal effects on images from the Railway Foreign Object Intrusion Sample Database are shown in Figure 10, where (a) shows the degraded image, (b) displays the ground truth (GT), (c) shows the result of MPRNet, (d) depicts TransWeather’s output, and (e) illustrates the effect of the WeatherClean algorithm in this section. The yellow boxes highlight areas with color distortion. The image processing includes both day and night scenes to assess the algorithms’ performance under different lighting conditions.

The rain removal visual effect on images from the Railway Foreign Object Intrusion Sample Database is shown in Figure 10i. Rain removal was carried out in both daytime and nighttime scenarios. The first row displays the daytime results, where all three algorithms effectively remove rain; however, MPRNet shows noticeable color distortion. In the second row, under nighttime conditions, both MPRNet and TransWeather exhibit color distortion, while our algorithm produces results closer to the ground truth.

The snow removal visual effect on images from the Railway Foreign Object Intrusion Sample Database is illustrated in Figure 10ii. Purple boxes highlight areas with significant noise, especially in the track region. In the first row, which shows daytime results, both MPRNet and TransWeather display unclear track areas with more noise, while NoiseWeather performs better at maintaining the clarity of the track region. In the second row, under nighttime conditions, the differences among the three algorithms are less pronounced.

The defogging visual effect on images from the Railway Foreign Object Intrusion Sample Database is shown in Figure 10iii. Green boxes indicate fog residue. Whether it is daytime or nighttime, both MPRNet and TransWeather still display significant amounts of fog that remain unremoved. Conversely, NoiseWeather performs well in defogging, effectively clearing the fog from the image without causing any color distortion.

In conclusion, these visual images further demonstrate that applying DNAM and MCL to image de-rain, de-snow, and defog tasks is highly effective.

### 4.4. Validation on Real Foggy Drone Images

To validate the effectiveness of the proposed model in real-world situations, this section tests it using actual foggy images taken by drones during early morning. We first show visual comparisons between original images and dehazed images, then perform basic object detection tasks to train and evaluate on datasets before and after dehazing, respectively. This demonstrates that the proposed dehazing method can effectively improve image quality and enhance the performance of subsequent visual tasks.

**Figure 11:**
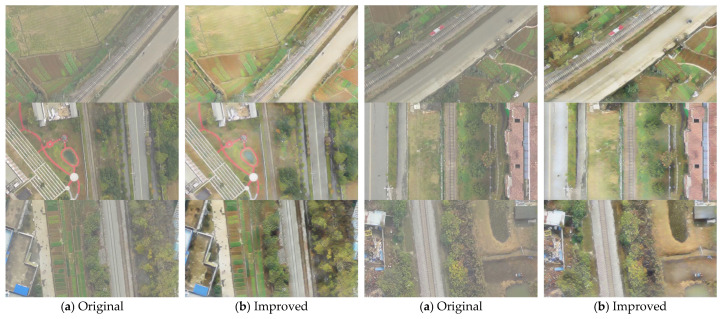
Visual comparison of defogging results on real-world drone images. (**a**) Original foggy drone images; (**b**) defogged images processed by WeatherClean.

Figure 11 shows the image comparison before and after dehazing with our model. It is clear that after dehazing, the overall image clarity improves significantly, fog occlusion is effectively removed, and the contours and details of distant targets are clearly restored. Especially under early morning fog conditions, the dehazed images not only keep the original color accuracy but also greatly enhance contrast and visibility, providing higher-quality input data for later computer vision tasks.

To thoroughly validate the performance boost of the proposed method on downstream visual tasks, we chose five leading object detection algorithms for comparison: YOLOv8 [31], YOLOv11 [32], DETR [33], Faster R-CNN [34], and CenterNet [35]. These algorithms represent different technical approaches in the current object detection landscape, including anchor-based detectors, query-based detectors, and keypoint-based detectors, which allow for a comprehensive assessment of the dehazing method’s applicability across various detection paradigms.

To ensure fairness and comparability of experimental results, we strictly controlled the experimental conditions: all algorithms were run on identical hardware with the same training hyperparameters, including learning rate, batch size, and optimizer configuration. The same random seed was used during training to guarantee result reproducibility, and identical evaluation protocols and data processing pipelines were followed during testing.

For evaluation metrics, we selected four core metrics in the object detection field: precision (P), recall (R), mean average precision (mAP@0.5), and mean average precision (mAP@0.5:0.95). Among them, precision measures the accuracy of detection results, representing the proportion of true positive samples among all samples detected as positive; recall measures the completeness of detection, representing the proportion of all true positive samples that are correctly detected; mAP@0.5 represents the mean average precision at an IoU threshold of 0.5, which is the most commonly used evaluation metric in object detection tasks; mAP@0.5:0.95 indicates the mean average precision over IoU thresholds from 0.5 to 0.95 (with a step size of 0.05), providing a more comprehensive assessment of detector performance under different localization accuracy requirements. This experiment focuses on a single-object category detection task, specifically “Person” detection.

The experimental results in Table 3 clearly show the significant performance improvements of the WeatherClean dehazing method on object detection. Comparing five mainstream object detection models on original foggy images and WeatherClean-processed images, all models demonstrated notable gains. In terms of mAP@0.5, YOLOv8 increased from 0.843 to 0.912 (8.2% rise), YOLOv11 from 0.812 to 0.875 (7.8% rise), DETR from 0.798 to 0.844 (5.8% rise), Faster R-CNN from 0.857 to 0.917 (7.0% rise), and CenterNet from 0.847 to 0.892 (5.3% rise). Notably, in precision, both YOLOv8 and Faster R-CNN achieved high precision levels of 0.934. These findings confirm that our WeatherClean model effectively removes fog interference from images, greatly enhances the performance of downstream visual tasks, and provides strong technical support for reliable drone operations under adverse weather conditions.

## 5. Conclusions

This study introduces a novel drone image rain–snow–fog removal framework called WeatherClean. The framework features a Dynamic Noise Adjustment Module and a Weather Complexity Adjustment Factor, which adaptively process rain, snow, and fog degradation of different intensities. It uses a hierarchical multi-scale cropping strategy to create local–global collaborative loss functions, improving the recovery of fine noise and edge details. Additionally, it develops a degradation synthesis method based on atmospheric scattering physical models to generate training samples that match real-world weather degradation patterns, helping to address data scarcity. This study effectively employs weather complexity adaptive adjustment and multi-scale cropping strategies, significantly enhancing image restoration performance under complex weather conditions.

However, this research still has some limitations. Limited by actual collection conditions, we mainly tested moderate-intensity foggy weather and have not yet verified the model’s robustness under extreme weather conditions such as heavy rain, blizzards, dense fog, and others. Future research will develop comprehensive datasets that include extreme weather conditions to thoroughly evaluate the dynamic adjustment capability of the WCAF under severe weather and assess the robustness of the proposed model.

## Figures and Tables

**Table 1:** WeatherClean component ablation study results.

Module	Snowy		Rainy		Foggy	
	PSNR	SSIM	PSNR	SSIM	PSNR	SSIM
baseline	27.91	0.9240	22.98	0.8107	18.63	0.8240
+DNAM	27.82	0.9266	25.64	0.8947	18.46	0.8233
+DNAM + MCL	28.12	0.9277	25.83	0.9107	28.44	0.9548

**Table 2:** Results of comprehensive comparative experiments.

Module	Snowy		Rainy		Foggy		FPS
	PSNR	SSIM	PSNR	SSIM	PSNR	SSIM	
MPRNet	28.17	0.7889	29.93	0.7797	12.44	0.7798	17.9
TransWeather	28.15	0.7758	29.67	0.7756	12.43	0.8233	**57.5**
Restormer	27.89	0.7623	28.45	0.7689	11.98	0.7956	28.3
Uformer	27.76	0.7589	29.32	0.7654	11.87	0.7892	25.7
All-in-One	**28.57**	0.7922	29.34	0.8023	14.23	0.8544	22.1
**WeatherClean**	28.49	**0.8024**	**30.05**	**0.8111**	**28.50**	**0.9548**	47.3

**Table 3:** Detection performance comparison of object detection models on original (left) and WeatherClean-Processed (right) foggy images.

Module	Original				Improved			
	P	R	mAP@0.5	mAP@0.5:0.95	P (↑)	R (↑)	mAP@0.5 (↑)	mAP@0.5:0.95 (↑)
YOLOv8	0.848	0.813	0.843	0.333	0.934	0.814	0.912	0.382
YOLOv11	0.826	0.796	0.812	0.331	0.863	0.832	0.875	0.369
DETR	0.821	0.756	0.798	0.317	0.861	0.768	0.844	0.344
Faster R-CNN	0.864	0.798	0.857	0.339	0.934	0.813	0.917	0.380
CenterNet	0.857	0.802	0.847	0.351	0.859	0.835	0.892	0.371

## Data Availability

For subsequent research, the dataset is not publicly available at this time.
